# Correlational links between professional identity and learning engagement among Chinese college students: the potential mediating role of positive emotions

**DOI:** 10.3389/fpsyg.2026.1935885

**Published:** 2026-10-29

**Authors:** Taiqi Zhan, Xiaolong Bi, Jingqiao Feng, Xinxin Zhang

**Affiliations:** 1 Hengyang Preschool Education College, Hengyang, China; 2 Faculty of Education, Bangkok Thonburi University, Bangkok, Thailand

**Keywords:** college students, learning engagement, mediating effect, positive emotions, professional identity

## Abstract

Against the backdrop of mass higher education in China, the quality of talent cultivation in local universities has become a major concern. The study was based on self-determination theory and social cognition theory, aimed to examine the relationship between professional identity and learning engagement among college students, as well as the mediating role of positive emotions in this relationship. The research sampled undergraduate students from four public universities in a central southern region of China. A total of 482 valid questionnaires were collected via an online survey. Descriptive statistics, independent samples t-tests, one-way ANOVA, Pearson correlation analysis, and hierarchical multiple regression combined with bias-corrected bootstrapping were employed for data analysis. The results indicated that participants reported moderate-to-high levels of professional identity, learning engagement, and positive emotions. Significant gender, grade, major, hometown, and family socioeconomic status differences were observed across the three constructs (all *p* < 0.001). Professional identity significantly and positively predicted both learning engagement and positive emotions. Positive emotions, in turn, positively predicted learning engagement and partially mediated the relationship between professional identity and learning engagement. The findings clarify the intrinsic mechanisms linking professional identity, positive emotions, and learning engagement, enrich empirical research on local university students, and provide practical implications for optimizing talent cultivation in regional higher education contexts.

## 1 Introduction

China’s higher education has entered the stage of popularization, and the quality of education has become the focus of social attention. As a core indicator to measure the learning process and educational effectiveness, learning engagement is the key to improving the quality of talent training (Reeve and Tseng, 2011; Fredricks et al., 2016; Wong and Liem, 2022). Its effectiveness in students ‘satisfaction with school (Luo et al., 2019), learning outcomes (Carini and Klein, 2006) and academic achievement (Collie et al., 2017; Yakup and Boyac, 2021) has received extensive attention. As an important pre-factor affecting learning motivation (Lee et al., 2020), which the level of professional identity is directly related to students ‘interest in learning and continuous input (Chen W. et al., 2023). However, at present, some college students lack rational planning due to professional choice, low professional recognition, and insufficient learning input, which restricts the effect of talent training. At the same time, positive emotions are found to be closely related to professional identity and learning engagement, which may play an intermediary role between them (Liu et al., 2023). From a theoretical perspective, these variable associations can be interpreted through Self-Determination Theory (Deci & Ryan) and Social Cognitive Theory (Bandura). Self-Determination Theory provides a well-established motivational framework to explain how identity-related need satisfaction shapes students’ affective experiences (Ryan Richard and Deci, 2016), while Social Cognitive Theory highlights affective resources as critical drivers of academic behavioural patterns. Reschly et al. (2008) conceptualized student engagement as a flourishing process supported by positive emotions and adaptive coping resources. Consistent with this view, emotional resources help individuals cope with academic stress; Meduri et al. (2026) further noted that positive affect and resilient coping are linked to reduced school-related adjustment risks. However, the relevant empirical research on local undergraduate college students in Hengyang City is still very scarce, and it is urgent to supplement the localized evidence.

Many Existing research has shown that professional identity, learning engagement, and positive emotions exhibit significant differences across background variables such as gender, grade, and major (Zhihai and Helin, 2006; Jinwei, 2018). However, conclusions remain inconsistent, and most studies focus on samples from national or first-tier cities, with insufficient targeted research on local universities in Hengyang. Academic emotions not only affect students’ cognitive processing, but also their learning motivation, use of learning strategies, and interest in learning (Wang and Holcombe, 2010; Wang and Eccles, 2013). Positive emotions can increase dopamine levels in the brain, thereby affecting long-term memory, working memory, and creative problem-solving (Li et al., 2020). At the same time, it can also help students maintain their interest in reading for a longer period of time (Ashby et al., 1999), improve their learning motivation and effort, and promote the flexible and creative use of their learning strategies (Ainley et al., 2005).

The study focused on undergraduate students in Central China, aiming to clarify the differential characteristics of professional identity, learning engagement, and positive emotions under different variables such as gender, grade, and major. It examines statistical associations of professional identity on learning engagement and positive emotions, and verifies correlational links of positive emotions on learning engagement and its mediating effect between professional identity and learning engagement. The study expanded the research framework of the relationship between professional identity and learning engagement by introducing positive emotions as a mediating variable, providing a new theoretical perspective for subsequent research. At the same time, it could provide targeted suggestions for local universities to optimize talent training programs, improve students’ professional identity and learning engagement levels, and help improve the quality of local higher education.

## 2 Methods

### 2.1 Ethics statement

The study was conducted based on Hengyang Preschool Teachers College. Ethical review and approval were not required for this research involving human participants in accordance with local legislation and institutional requirements. All participants in this study provided online informed consent forms before completing the questionnaire survey. Participants were informed that participation is entirely voluntary and they can withdraw at any time without penalty. All responses are anonymous to ensure data confidentiality. This study did not collect any personal identification information.

### 2.2 Participants

The study recruited participants through the Tencent Questionnaire.^1^ A survey of college students in four public universities in Central Ching, Inland area of China in 2025 was conducted. One hundred seventy students were selected from each school to conduct a questionnaire survey. A total of 562 questionnaires were completed. The inclusion criteria were full-time undergraduate students who voluntarily participated and provided informed consent, and who completed at least 90% of the questionnaire items. Exclusion criteria included students on leave or long-term academic suspension, as well as questionnaires with excessive missing data or clear patterns of careless responses. Ultimately, 482 valid questionnaires were obtained. All participants were Chinese nationals, comprising 214 boys and 268 girls. The mean age of participants was 19.27 ± 1.32 years, ranging from 18 to 22 years. In terms of academic year distribution: freshmen accounted for 30.9% (149 individuals), sophomores for 29.7% (143 individuals), juniors for 21.0% (101 individuals), and seniors for 18.5% (89 individuals). Participants represented five majors: humanities and history (29.5%), science and engineering (33.8%), economics and management (25.5%), and arts (11.2%). The same inclusion and exclusion criteria applied as previously stated.

### 2.3 Research tools

The scale contents of the study are divided into the College Student Background Scale, College Student Professional Identity Scale, College Student Learning Engagement Scale, and College Student Positive Emotion Scale. In addition to the background variable of college students, the other three parts of the questionnaire were scored using a Likert five points scale, with 5, 4, 3, 2, and 1 representing “strongly agree,” “somewhat agree,” “average,” “disagree,” and “strongly disagree,” respectively. The higher the professional identity, learning engagement, and positive emotions of college students, the lower the opposite.

The background variables of college students are divided into five aspects: gender (male, female), grade (freshman, sophomore, junior, and senior), major (literature and history, science and engineering, economics and management, art), place of origin (urban, rural) and family economic status (poor, general, good). The College Student Professional Identity Scale comes from the College Student Professional Identity Scale developed by Carvalho et al. (2021), which includes four dimensions: cognition, emotion, behavior, and appropriateness (Supplementary Table S1). The revised version of the College Student Learning Engagement Scale (UWES-S) by Carmona-Halty et al. (2019) included three dimensions: motivation, energy, and focus (Supplementary Table S2). The positive emotion scale for college students mainly refers to Watson et al. (1988) “Positive and Negative Emotion Scale” (Supplementary Table S3), and only the positive emotion scale part of the scale is taken.

### 2.4 Statistical analysis

The study used multiple statistical methods to conduct data analysis. Firstly, descriptive statistics are used to calculate the mean, standard deviation, and proportion of each variable dimension, in order to clarify the overall distribution characteristics of college students’ professional identity, learning engagement, and positive emotions. Secondly, independent sample *t*-test was used to analysis the inter group differences of background variables such as gender and place of origin in each research variable. Thirdly, the differential effects of grade, major, and family economic status were tested through one-way ANOVA. For indicators with significant differences, Scheffe *post hoc* tests were further used to determine the mean differences between groups. Fourthly, Pearson product difference correlation analysis is used to explore the degree of correlation between variables, and the correlation level is defined according to the correlation coefficient classification standard of Rowat et al. (2017). Finally, hierarchical multiple regression combined with bias-corrected bootstrapping was performed to examine correlational patterns among variables. Demographic covariates (gender, grade, major, place of origin, family socioeconomic status) were entered in Block 1. Professional identity was entered in Block 2 as the independent variable to examine its statistical association with learning engagement and positive emotions. Positive emotions (mediator) were added in Block 3. To formally test the mediating potential, bootstrapping with 5,000 resamples was implemented to estimate the total effect, direct effect, and indirect effect, together with bias-corrected 95% confidence intervals (CIs). A non-zero indirect effect (i.e., 95% CI does not contain zero) was regarded as evidence supporting mediating potential. All visual images were completed using R software (v4.3.2).

## 3 Results

### 3.1 Descriptive statistics of college students’ background variables

As shown in Figure 1, at the gender level, the proportion of female students in the sample was relatively high. In terms of grade distribution, freshmen and sophomores dominate, while the proportion of junior and senior students in the sample was relatively low, which distribution feature was mainly due to the fact that senior students participate more in extracurricular internships, job hunting, and other school affairs. In terms of majors, the sample size of art majors was relatively small, which was in line with the small enrollment scale of art majors in comprehensive applied undergraduate colleges. Under the dimension of origin, the proportion of rural students was higher than that of urban students, which was closely related to the population distribution characteristics of China’s rural population with a large population base. In terms of family economic status, the sample group was mainly composed of families with moderate to above average economic levels, and the proportion of families with superior economic conditions was also at a relatively high level. The result intuitively reflects the social development status of steady economic development and continuous improvement of residents’ quality of life in China.

**Figure 1:**
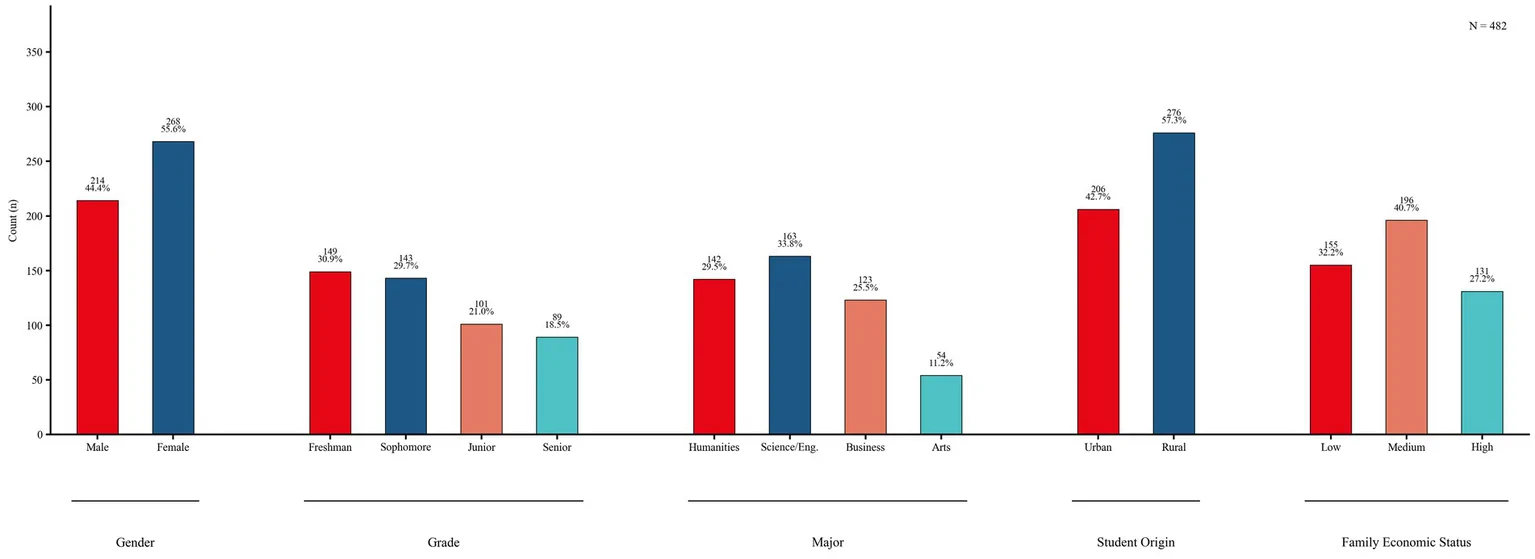
Analysis of questionnaire survey sample.

### 3.2 The current situation of college students ‘professional identity, learning engagement and positive emotions

The study found that the overall mean of professional identity was 3.88 (SD = 0.38) across the cognitive dimension (M = 3.63, SD = 0.60), affective dimension (M = 3.86, SD = 0.47), behavioral dimension (M = 4.04, SD = 0.48), and appropriateness dimension (M = 3.96, SD = 0.64). The score range for each dimension was 3.63 to 4.04, indicated that the overall professional identity of the sample college students was above the medium level (Figure 2A; Table 1). It was worth noting that the behavioral dimension scores the highest, while the cognitive dimension scores the lowest, which suggested that college students possess good professional behavioral initiative and were able to actively complete professional learning tasks. However, their understanding of the essence and connotation of their majors and the development trends of the industry was insufficient, and their professional cognitive system was not yet perfected.

**Figure 2:**
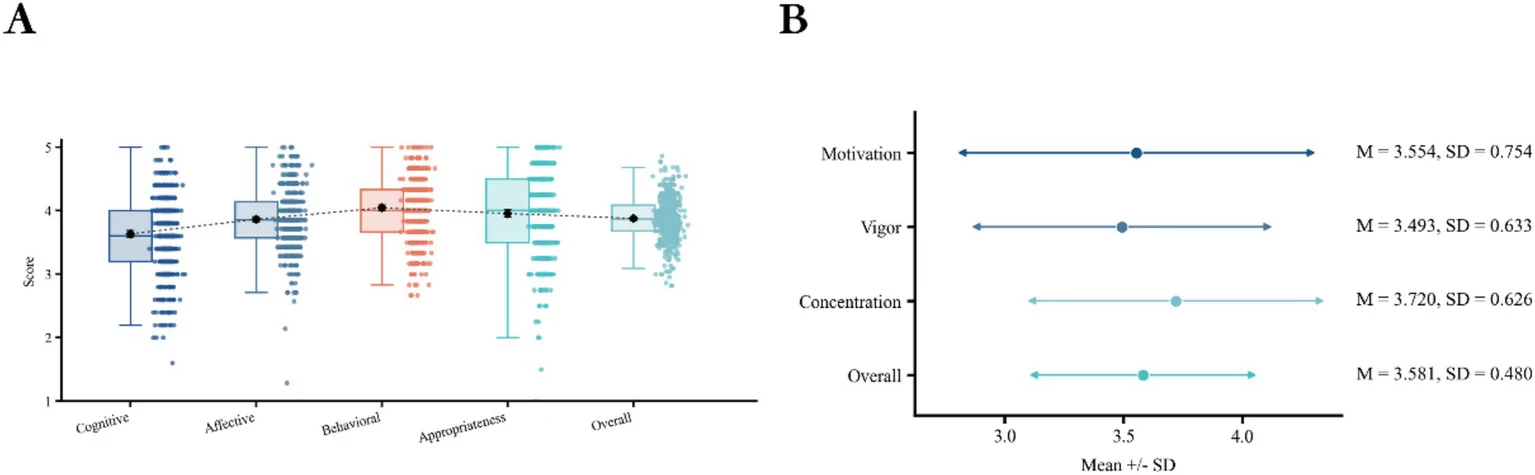
Descriptive statistics of professional identity and learning engagement. **(A)** Descriptive statistics of professional identity; **(B)** Descriptive statistics of learning engagement.

**Table 1:** The average value and standard deviation of each dimension of college students’ professional identity.

Dimensions	Number of samples	Mean value	Standard deviation
Cognitive	482	3.634	0.598
Emotionality	482	3.863	0.466
Behavioral	482	4.043	0.482
Appropriateness	482	3.955	0.639
Professional identity	482	3.877	0.377
Positive emotions	482	3.682	0.576

In terms of learning engagement, the distribution of sample dimensions and overall scores range from 3.49 to 3.72 (Figure 2B). Although the overall score is in the upper middle range, the overall score was relatively low, indicated that the level of college students’ learning engagement still needed further improvement. In addition, the average positive emotion of college students was 3.68 (SD = 0.58, Table 1), which was also at a medium to high level and had not yet reached the excellent threshold.

### 3.3 Gender differences

In terms of professional identity, there were significant gender differences in emotionality (t = 4.28, *p* < 0.001), appropriateness (t = 2.66, *p* < 0.01), and overall professional identity (t = 3.451, *p* < 0.01), with male samples scoring significantly higher in emotionality, appropriateness, and overall professional identity than females (Figure 3A). In terms of learning engagement, there were significant differences in energy (t = 5.55, *p* < 0.001), focus (t = 4.91, *p* < 0.001), and overall learning engagement (t = 4.15, *p* < 0.001). Male students had significantly better levels of engagement than female students (Figure 3B). In addition, there was a significant gender difference in positive emotions (t = 8.71, *p* < 0.001), with males scoring higher in positive emotions (Figure 3C). Overall, male college students exhibit superior levels in emotional identification, adaptation perception, learning engagement, and positive emotions.

**Figure 3:**
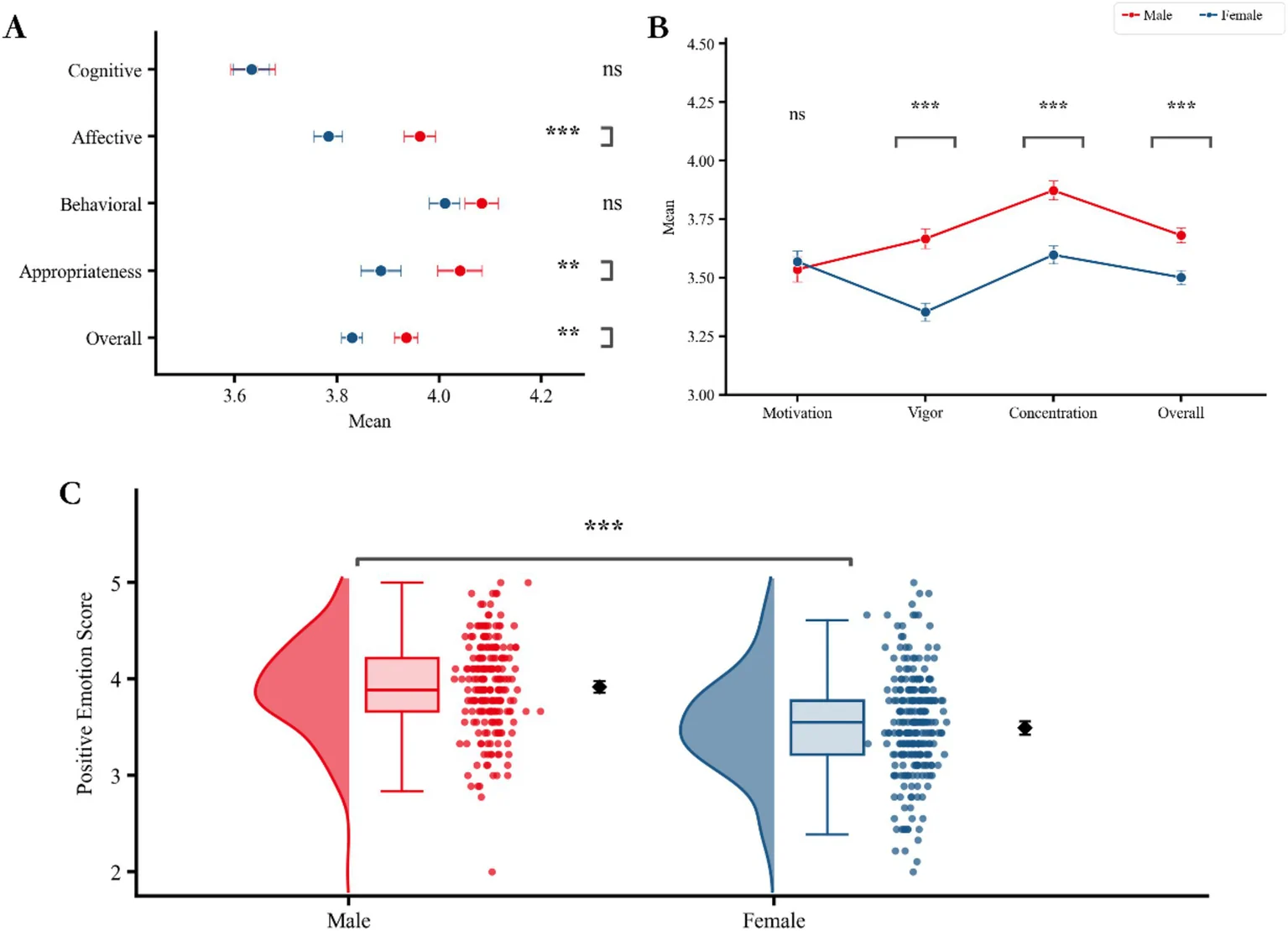
Analysis of gender differences in professional identity, learning engagement, and positive emotions among college students. **(A)** Analysis of differences in professional identity among college students of different genders; **(B)** Analysis of differences in learning engagement among college students of different genders; **(C)** Analysis of differences in positive emotions among college students of different genders.

### 3.4 Grade differences

The study found significant grade differences in various dimensions of professional identity, with cognitive (*F* = 6.48, *p* < 0.001), affective (*F* = 6.54, *p* < 0.001), behavioral (*F* = 4.49, *p* < 0.01), appropriateness (*F* = 6.02, *p* < 0.001), and overall professional identity (*F* = 8.51, *p* < 0.001) all reaching significant levels, and junior and senior students having better professional cognitive levels than sophomore students; Freshman students had significantly higher levels of emotional, behavioral, appropriateness, and overall identification compared to senior students (Figure 4A). In terms of learning engagement, there were significant grade differences in each dimension, with *F*-values for motivation, energy, focus, and overall engagement being 5.60, 10.35, 10.07, and 5.14, respectively (*p* < 0.01). Third year students had the strongest learning motivation, while first-year students perform the best in energy, focus, and overall engagement (Figure 4B). In addition, there was a significant grade difference in positive emotions (*F* = 41.27, *p* < 0.001), and first-year students scored the highest in positive emotions (Figure 4C).

**Figure 4:**
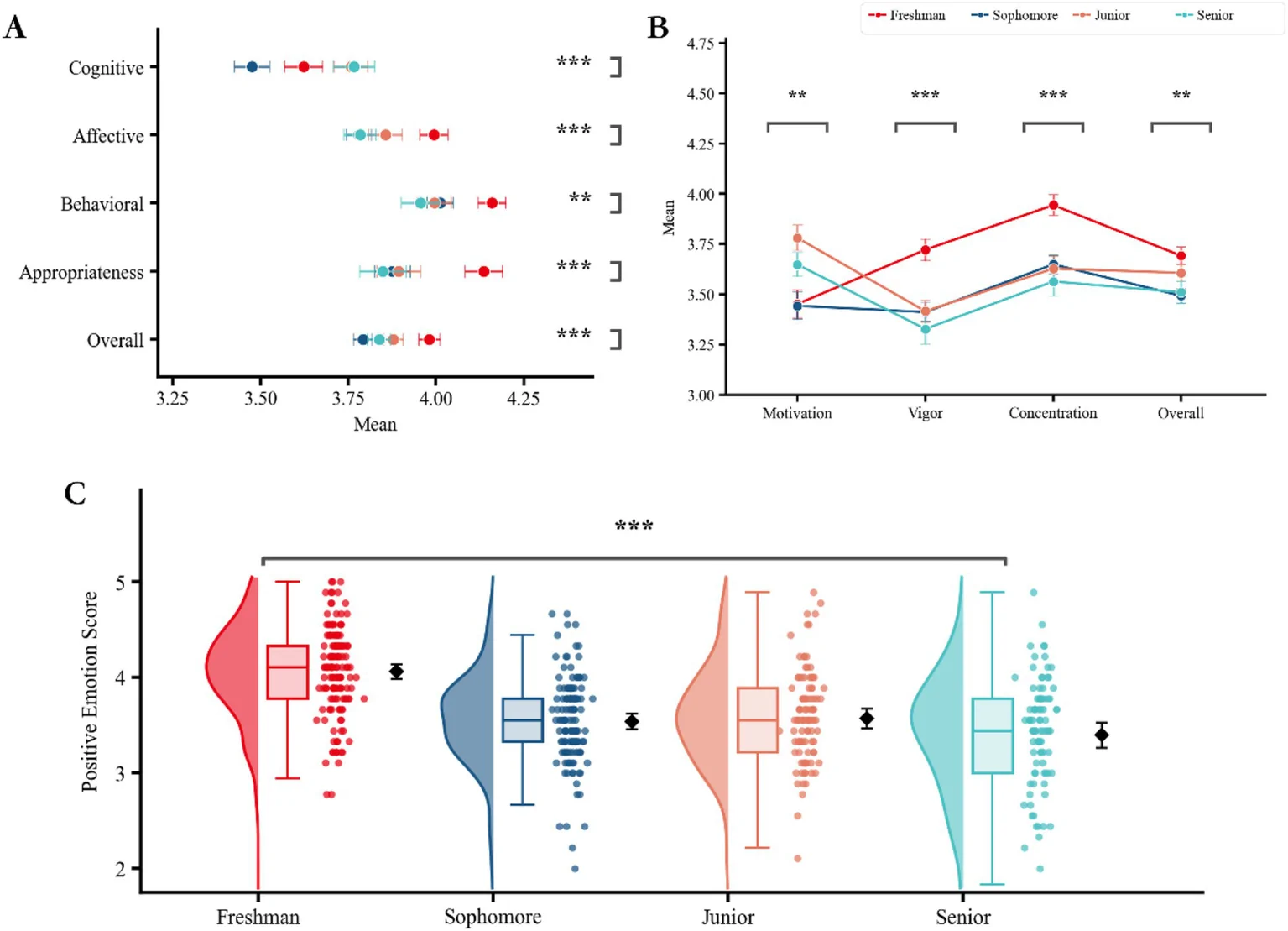
Analysis of differences in college students’ professional identity, learning engagement, and positive emotions across grades. **(A)** Analysis of differences in professional identity among college students of different grades; **(B)** Analysis of differences in learning engagement among college students of different grades; **(C)** Analysis of differences in positive emotions among college students of different grades.

### 3.5 Professional differences

At the level of professional identity, there were significant differences in emotional, behavioral, appropriateness, and overall professional identity (*F* = 5.37–9.80, *p* < 0.01). Students majoring in literature and history had significantly higher emotional and overall professional identity than students majoring in science and engineering, and students majoring in literature and history also had better behavioral and appropriateness dimensions than students majoring in science and engineering (Figure 5A). In terms of learning engagement, there were significant professional differences in motivation, energy, focus, and overall engagement (*F* = 2.90 ~ 5.23, *p* < 0.05), with management students having higher learning motivation than art students; Students majoring in literature, history, and management have significantly better levels of energy, focus, and overall investment than students majoring in science and engineering (Figure 5B). In addition, there were significant professional differences in positive emotions (*F* = 11.75, *p* < 0.01), with science and engineering students scoring the lowest in positive emotions (Figure 5C).

**Figure 5:**
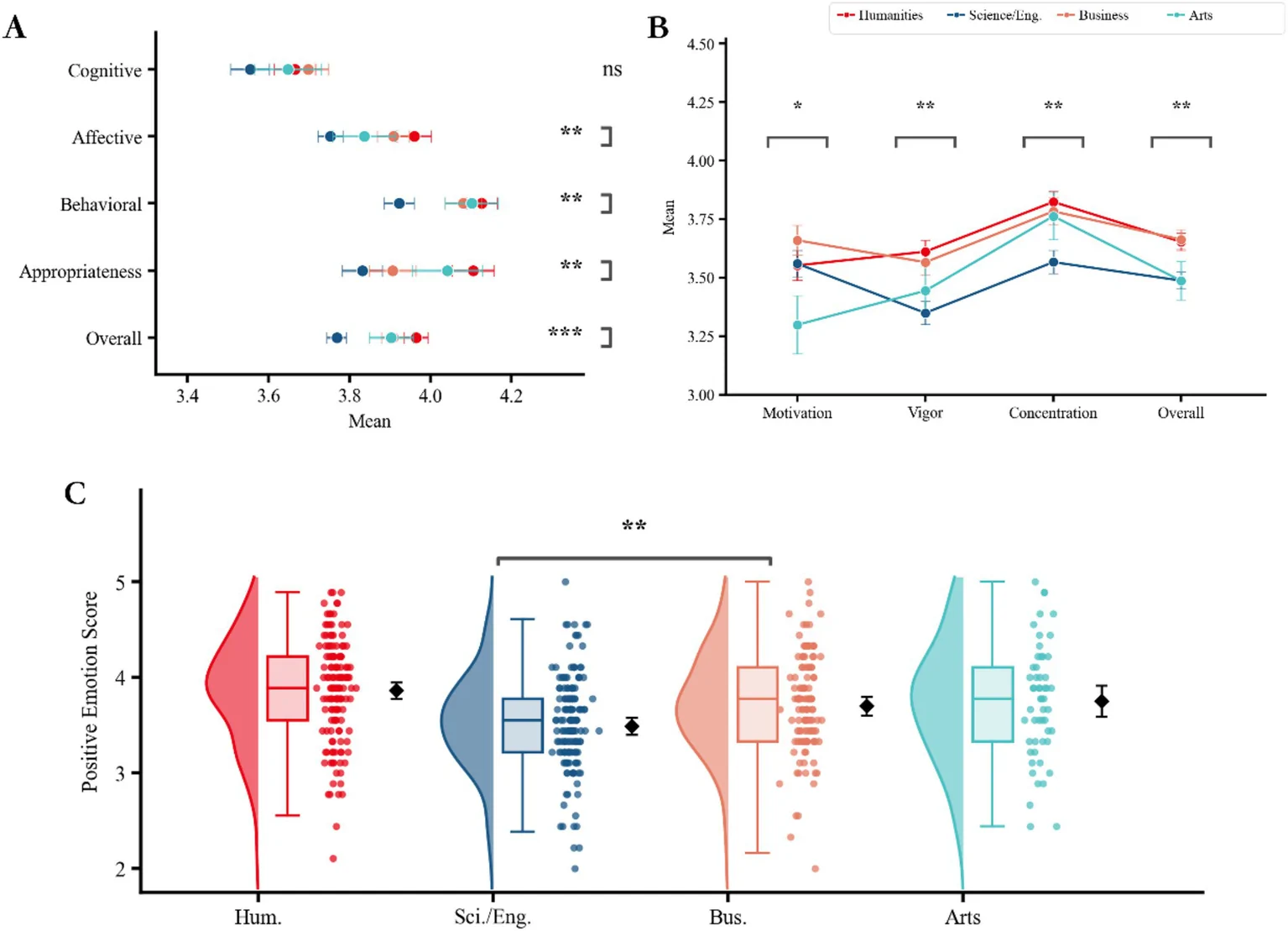
Differences in professional identity, learning engagement, and positive emotions among college students across different majors. **(A)** Differences in professional identity among college students from different majors; **(B)** Differences in learning engagement among college students of different majors; **(C)** Differences in positive emotions among college students of different majors.

### 3.6 Differences in place of origin

The dimensions of emotionality (t = 3.74, *p* < 0.001), appropriateness (t = 2.69, *p* < 0.01), and overall professional identity (t = 2.74, *p* < 0.01) had all reached a statistically significant level, and urban students scored significantly higher than rural students in the dimensions of emotionality, appropriateness, and overall professional identity (Figure 6A). Urban students scored significantly higher than rural students in terms of energy, focus, and overall learning engagement (*p* < 0.01, Figure 6B). In addition, the positive emotion scores of urban students were significantly higher than those of rural students (t = 5.69, *p* < 0.001, Figure 6C).

**Figure 6:**
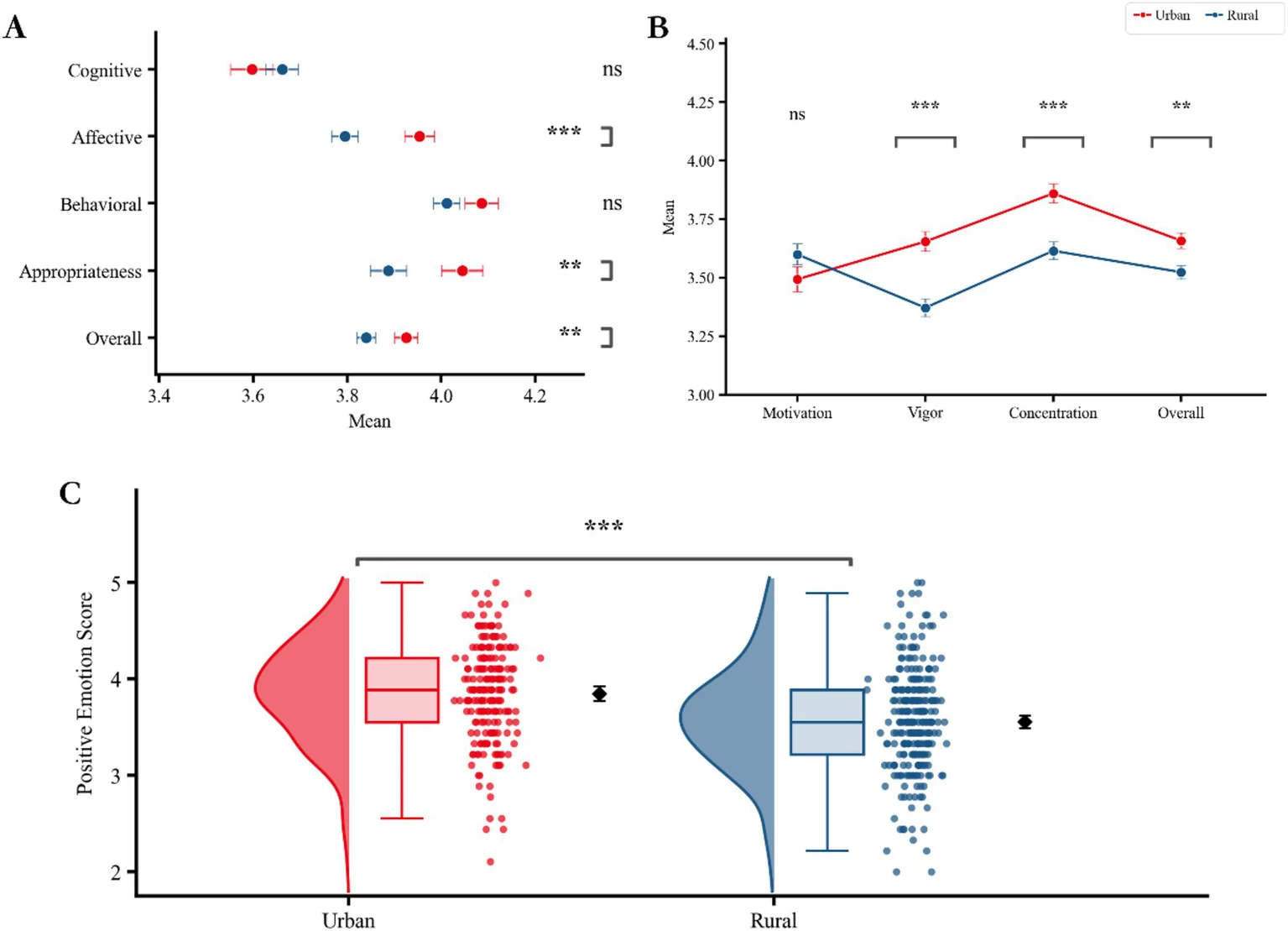
Differences in professional identity, learning engagement, and positive emotions among college students in different regions of origin. **(A)** Differences in professional identity among college students from different origins; **(B)** Differences in learning engagement among college students from different origins; **(C)** Differences in positive emotions among college students from different origins.

### 3.7 Differences in family economic status

The three dimensions of emotional, behavioral, and appropriateness all showed that students from families with poor economic conditions scored significantly higher than students from families with average economic conditions (*p* < 0.01), while the overall score of professional identity was highest among students from families with poor economic conditions (Figure 7A). In terms of learning engagement, students with average economic conditions scored significantly lower in terms of energy, focus, and overall learning engagement compared to students from economically disadvantaged families (*p* < 0.01, Figure 7B). In addition, the *F*-value of positive emotions among college students from different economic backgrounds was 19.73 (*p* < 0.001), reached a statistically significant level. Students from average economic backgrounds scored the lowest in positive emotions (Figure 7C).

**Figure 7:**
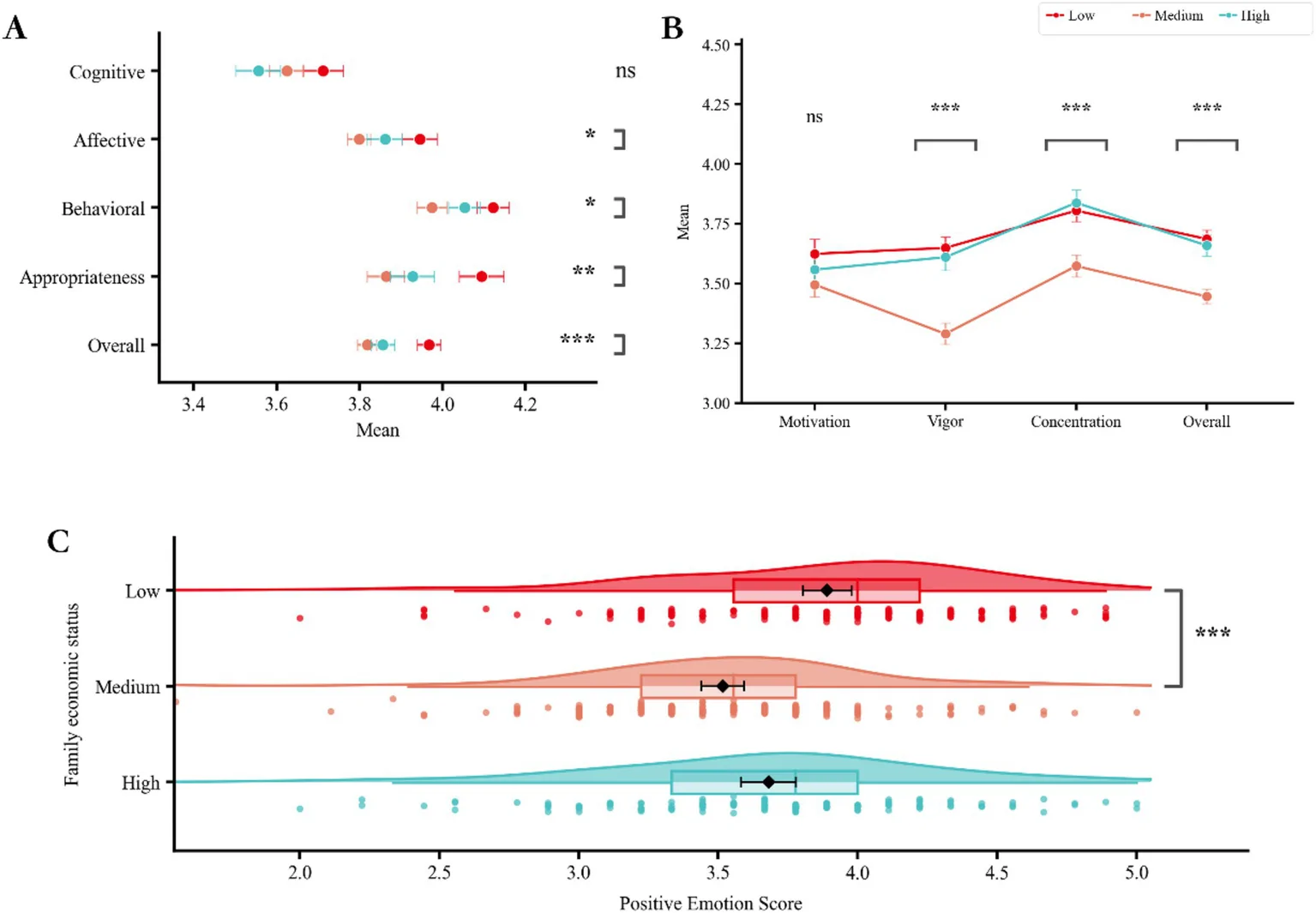
Differences in professional identity, learning engagement, and positive emotions among college students in different family economic conditions. **(A)** Differences in professional identity among college students from different family economic situations; **(B)** Differences in learning engagement among college students from different family economic conditions; **(C)** Differences in positive emotions among college students from different family economic backgrounds.

### 3.8 Correlation analysis between professional identity, learning engagement, and positive emotions

The study found that the cognition of professional identity was only positively correlated with the motivation of learning engagement and the dimension of concentration. The dimensions of affective, behavioral and appropriateness were significantly positively correlated with the three dimensions of learning engagement (Table 2). There was a significant low positive correlation between each dimension of professional identity and positive emotions (Table 3). In the dimension of learning engagement, motivation had a low positive correlation with positive emotions, while energy and concentration had a significant positive correlation with positive emotions (Table 4). On the whole, there was a significant positive correlation between professional identity, learning engagement and positive emotion. The overall correlation coefficient between professional identity and learning engagement was 0.45, the overall correlation coefficient between professional identity and positive emotion was 0.43, and the overall correlation coefficient between learning engagement and positive emotion was 0.46 (*p* < 0.001).

**Table 2:** Correlation analysis between professional identity and learning engagement.

Dimensions	Cognitive	Emotionality	Behavioral	Appropriateness	Motivation	Energy	Focus
Cognitive	1						
Emotionality	0.212***	1					
Behavioral	0.095*	0.233***	1				
Appropriateness	0.121**	0.203***	0.351***	1			
Motivation	0.415***	0.101*	0.101*	0.092*	1		
Energy	0.053	0.206***	0.276***	0.284***	0.156**	1	
Focus	0.133**	0.252***	0.255***	0.333***	0.128**	0.543***	1

*p < 0.05, **p < 0.01, ***p < 0.001.

**Table 3:** Correlation analysis between professional identity and positive emotion.

Dimensions	Cognitive	Emotionality	Behavioral	Appropriateness	Positive emotions
Cognitive	1				
Emotionality	0.212***	1			
Behavioral	0.095*	0.233***	1		
Appropriateness	0.121**	0.203***	0.351***	1	
Positive emotions	0.173***	0.318***	0.272***	0.332***	1

*p < 0.05, **p < 0.01, ***p < 0.001.

**Table 4:** Correlation analysis between learning engagement and positive emotions.

Dimensions	Motivation	Energy	Focus	Positive emotions
Motivation	1			
Energy	0.156**	1		
Focus	0.128**	0.543***	1	
Positive emotions	0.135**	0.444***	0.461***	1

*p < 0.05, **p < 0.01, ***p < 0.001.

### 3.9 Regression and mediation analysis of professional identity, learning engagement, and positive emotions

The regression analysis results indicated that professional identity had a significant positive predictive effect on positive emotions (*β* = 0.351) and learning engagement (*β* = 0.433) (Figures 8A,B), that is, the higher the individual’s level of professional identity, the better their learning level, and the higher their positive emotions. Positive emotions could significantly and positively predict learning engagement (*β* = 0.489, *p* < 0.001, Figure 8C), meaning that in this sample, college students reporting higher positive emotions tended to report higher learning engagement. After incorporating the mediator variable (positive emotions), the standardized regression coefficient of professional identity on learning engagement decreased from 0.433 to 0.309, and this direct effect still had statistical significance (*p* < 0.001, Figure 8D), Bootstrap-based mediation results (5,000 resamples) yielded the following standardized effect estimates: total effect = 0.433, direct effect = 0.309, indirect effect = 0.124, bias-corrected 95% CI [0.082, 0.175]. Since the 95% confidence interval for the indirect effect did not include zero, the results supported partial correlational mediating potential of positive emotions in the association between professional identity and learning engagement.

**Figure 8:**
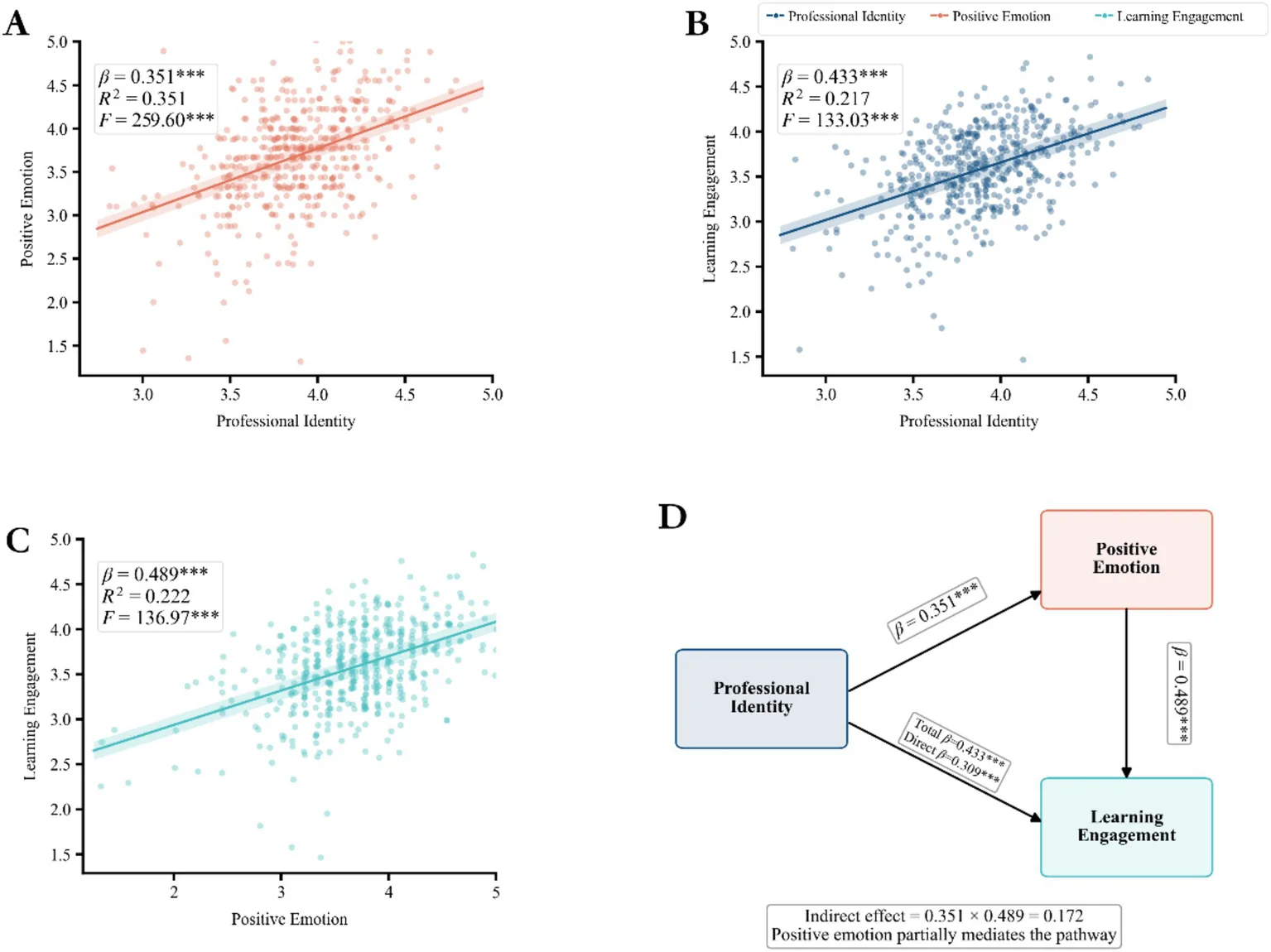
Regression analysis and mediation path analysis. **(A)** Professional identification predicts positive emotions; **(B)** Professional identification predicts learning engagement; **(C)** Positive emotions predict learning engagement; **(D)** Analysis of intermediary path.

## 4 Discussion

The study found that the dimensions and overall scores of college students’ professional identity ranged from 3.63 to 4.04, which was above average and consistent with the research results of Qin et al. (2024), slightly higher than the research results of Lee and Kim (2011). The cognitive professional identity score was the lowest, indicated that students do not have a deep understanding of their major, while the behavioral professional identity score was the highest, reaching a good level, indicated that students perform well in practical actions. The scores of each dimension and the whole of students’ learning engagement were between 3.49 and 3.72. The scores of each dimension and the whole of students’ learning engagement were not very high, which belongs to the upper middle level, indicated that college students need to strengthen their learning engagement (Wang et al., 2023). The average value of each dimension and the whole of students’ positive emotions was 3.68, which was above the middle level, indicated that students ‘emotions were not very high and need to be strengthened (Klutsey and Mahama, 2025). It should be noted that group-difference statistical outcomes reported in the Results section represent empirical associations observed within this sample. Interpretations for why these background-related differences emerge are post-hoc speculative accounts and cannot be treated as definitive conclusions, given the cross-sectional nature of this study. No causal inferences can be drawn from the present dataset.

The gender difference was that the level of professional identity of boys were higher than that of girls, one potential interpretation was that this pattern might be related to the fact that boys were more inclined to choose interest-oriented majors, while girls were vulnerable to social identity and chose majors with low adaptability (Wigfield, 1994). In terms of grade differences, the professional cognitive dimension score of senior students was significantly higher than that of junior students. It was speculated that this might correlate with the deepening of learning, students’ understanding of the profession continues to deepen; the freshmen had a higher level of recognition in the dimensions of emotion and behavior, which might be associated with the freshmen’s initial expectation of the major and their stronger enthusiasm for action (Chen et al., 2025). In terms of major types, the professional identity of science and engineering students was significantly lower than that of literature and history management students, a tentative explanation could be that this might relate to the high theoretical and technical threshold of science and engineering majors, and the weak professional identity of some students (Stets et al., 2016; Ovink et al., 2024). The difference in the source of students was that the level of professional identity of urban students was higher than that of rural students. This pattern mighty correlate with the richer educational resources and broader professional vision of urban students. Additionally, students with poor family economic conditions showed higher professional identity. It is possible that such students had stronger expectations and motivation for university and professional learning, and hope to change their own situation through professional learning (Zeng et al., 2022).

There were significant differences between college students’ learning engagement and positive emotions in background variables such as gender, grade, major, place of origin and family economic status, which was basically consistent with the existing research conclusions at home and abroad (Geng et al., 2024; Jiang et al., 2026). The gender difference was that the level of learning engagement and positive emotions of boys was significantly higher than that of girls, which might correlate with the learning adaptation model and self-expression motivation of boys at the university level (Qu and Bhatta, 2025). In terms of grade differences, older students scored higher in the cognitive dimension of learning engagement, this might be associated with as their learning progresses, their learning methods and sense of urgency gradually increase; Freshman students performed the best in other dimensions of learning engagement and positive emotions, potentially linked to their learning inertia and expectations for university during the freshman stage make them emotionally charged and energetic (Li and Xue, 2023; Hu et al., 2024). In terms of major types, the learning engagement and positive emotions of science and engineering students were significantly lower than those of other majors. One potential interpretation is that the depth and difficulty of the course could easily lead to learning burnout and negative emotions (Ippolito and Kingsbury, 2024). The difference of students’ origin showed that the level of learning engagement and positive emotion of urban students was generally higher than that of rural students, which might correlate with their richer educational resources and more optimistic attitude (Zhao et al., 2021). Beyond this, family socioeconomic status may be correlated with variations in students’ learning engagement and emotional states. Students facing financial hardship often showed higher levels of academic resilience and more positive affect (Wu et al., 2022; Shariq et al., 2026). These observed group disparities reflect correlational patterns, and might offer tentative insights for colleges and universities to implement targeted education.

The gender difference was that the positive emotion level of boys was significantly higher than that of girls, which might be related to the stronger self-expression motivation and competition consciousness of boys when they first enter university (Rehman et al., 2018). In terms of grade differences, freshmen had the highest level of positive emotions, potentially associated with the freshness and expectation of college life in the freshmen stage made their emotions rise (Alarcón et al., 2020). In terms of major types, the positive emotions of science and engineering students were significantly lower than those of other majors, which might be correlate with the fact that the high difficulty and challenge of the course could easily lead to academic pressure and negative emotions (Pester et al., 2023). The difference in the source of students showed that the level of positive emotions of urban students was generally higher than that of rural students, which might be linked to their richer educational resources and more optimistic growth environment (Wan et al., 2025). It was worth noting that students with poor family economic conditions had a higher level of positive emotions. It was tentatively proposed that such students were full of yearning for hard-earned university opportunities and showed stronger emotional resilience and positive mentality. This coincides with the theory of Gasiorowska et al. (2025) ‘Healthy emotions are a strong resilience factor against social and economic adversity and transcend the protective effect of perceptual control’. These observed group differences reveal correlational associations among individual background factors and positive emotions, and might provide tentative basis for targeted mental health education in colleges and universities.

The study verified significant correlations among college students’ professional identity, positive emotions and learning engagement, with positive emotions serving as a partial mediator. Specifically, professional identity was significantly and positively correlated with learning engagement across all dimensions and the overall scale (*r* = 0.450, *p* < 0.001). Regression analyses further revealed that professional identity exerted a significant predictive effect on learning engagement (*β* = 0.433, *p* < 0.001). These findings suggested that higher levels of professional identity correspond to greater learning engagement among students, which aligns with previous research reported by Yu et al. (2021) and Vallerand et al. (1992). Secondly, there was a significant low positive correlation between professional identity and positive emotions in all dimensions, and the overall positive correlation was moderate (*r* = 0.430, *p* < 0.001), and it could significantly predict positive emotions (*β* = 0.351, *p* < 0.001) Furthermore, positive emotions were significantly positively correlated with all dimensions of learning engagement, and the overall correlation was positive (*r* = 0.458, *p* < 0.001), and had a significant predictive effect on learning engagement (*β* = 0.489, *p* < 0.001), which was consistent with Frenzel et al. (2007) ‘control-value theory ‘of academic emotions. Finally, the mediating effect test showed that the standardized regression coefficient of professional identity on learning engagement decreased from 0.433 to 0.309 (still significant) after the inclusion of positive emotions, indicating that positive emotions played a partial mediating role between the two, cross-sectional mediation results demonstrate correlational pathways, that was professional identity could indirectly promote learning engagement by enhancing positive emotions, which provided an empirical basis for understanding the chain mechanism of the three (Chen Q. et al., 2023; Liu et al., 2023b).

Although the present study uncovered meaningful associations among college students’ professional identity, positive emotions, and learning engagement and confirmed the partial mediating role of positive emotions, several limitations should be acknowledged. As a cross-sectional study, it cannot establish causal relationships among the three core variables despite regression-based mediation testing; longitudinal or experimental designs are required to validate directional paths in future research. Besides, all data rely on self-report questionnaires, which may lead to common-method bias, so future investigations may integrate objective indicators such as academic performance records and teacher-rated learning engagement to improve measurement robustness. Furthermore, participants were solely recruited from comprehensive applied undergraduate colleges, with over-represented freshmen and sophomores and under-represented seniors owing to internships and job-hunting activities, which limits the generalizability of findings to research-oriented universities, vocational colleges, and senior undergraduates. Finally, key individual information including age was not collected for further subgroup analysis, and potential confounding factors such as coping style and social support were excluded from the current model, which deserve consideration in follow-up work.

## 5 Conclusion

The study explored group differences in professional identity, learning engagement, and positive emotions across demographic backgrounds, alongside correlational interrelationships among these three constructs among Chinese undergraduate students. Based on cross-sectional questionnaire data, significant demographic group differences were observed for professional identity, learning engagement, and positive emotions. Both professional identity and positive emotions represented significant positive correlates of learning engagement; positive emotions demonstrated partial correlational mediating potential in the association between professional identity and learning engagement. Given the cross-sectional design, causal inferences cannot be drawn from the present dataset. Informed by these correlational findings, higher-education institutions may offer diversified major-related guidance, optimize instructional approaches, and expand practical training and academic-exchange opportunities to foster students’ professional identity. For individual students, active career-planning participation, autonomous learning development, practical-experience engagement, and enhanced major-relevant communication can help strengthen professional-identity perceptions and learning motivation. Future research should broaden geographic and institutional sampling, adopt longitudinal or mixed-methods designs, incorporate qualitative inquiry, and examine additional mediating or moderating variables to deepen understanding of how professional identity may correlate with learning engagement.

## Data Availability

The original contributions presented in the study are included in the article/Supplementary material, further inquiries can be directed to the corresponding author.

## Footnotes

1
https://wj.qq.com/login.html
